# A qualitative study to examine meaningful change in physical function associated with weight-loss

**DOI:** 10.1007/s11136-022-03191-2

**Published:** 2022-07-22

**Authors:** Jiat-Ling Poon, Chris Marshall, Chloe Johnson, Hannah C. Pegram, Maile Hunter, Hongjun Kan, Nadia N. Ahmad

**Affiliations:** 1 Value, Evidence, and Outcomes Center of Innovation, Eli Lilly and Company, Indianapolis, IN USA; 2 Clinical Outcomes Assessment, Clarivate Analytics, London, UK; 3 Formerly of Clinical Outcomes Assessment, Clarivate Analytics, Nashville, TN USA; 4 Value, Evidence, and Outcomes, Eli Lilly and Company, Indianapolis, IN USA; 5 Lilly Diabetes, Eli Lilly and Company, Indianapolis, IN USA

**Keywords:** Adults, Obesity, Physical activity, Quality of life

## Abstract

**Purpose:**

This study explored perceptions of meaningful weight-loss and the level of change on two patient-reported outcome (PRO) measures, the 36-item Short Form Health Survey® [SF-36v2®] and Impact of Weight on Quality of Life Lite-Clinical Trials^©^ [IWQOL-Lite-CT^©^], that individuals living with overweight or obesity consider to be meaningful and indicative of treatment success.

**Methods:**

Thirty-three qualitative interviews were conducted in the US with adults living with overweight or obesity. Concept elicitation explored perceptions of minimally important/meaningful weight-loss using open-ended questions. Cognitive debriefing was used to understand thresholds for meaningful change on both measures.

**Results:**

Most participants (*n* = 23/33) expected a 5% total body weight-loss to yield some benefit in physical functioning, while all participants expected a 10% weight-loss to provide a meaningful and noticeable improvement in their physical functioning. Participants indicated that an item-level 1-point score change on each measure would represent a noticeable improvement in physical functioning and indicate treatment success.

**Conclusions:**

Participants expected moderate weight-losses to be noticeable, with ≥ 10% weight-loss yielding the most consistent results. The findings suggested that both measures provide strong opportunity to demonstrate treatment benefit in relation to physical functioning as a small change on the response scale would represent a noticeable improvement in participants’ daily lives.

**Supplementary Information:**

The online version contains supplementary material available at 10.1007/s11136-022-03191-2.

## Plain English summary

Previous studies in chronic weight-management have shown improvement in physical function after weight-loss, however, it is not clear if the level of change achieved is meaningful to patients. This study aimed to address this gap in published literature by exploring meaningful weight-loss from a patient perspective. Specifically, the level of change that people living with obesity consider to be meaningful and indicative of treatment success on two patient-reported outcome measures was discussed (the 36-item Short Form Health Survey® [SF-36v2®] and Impact of Weight on Quality of Life Lite-Clinical Trials© [IWQOL-Lite-CT©]). Our manuscript presents the findings from interviews involving thirty-three adults in the US living with obesity. Most participants (*n* = 23/33) expected that a 5% total body weight-loss would lead to improvements in physical functioning, while all participants felt that a 10% total body weight-loss would lead to a noticeable improvement in physical functioning. Participants suggested that a 1-point score change on the PRO measures would be a noticeable improvement and indicate that a treatment had been successful. Our findings add to the existing literature by providing a deep understanding and rich, qualitative insights into how individuals’ contextualize meaningful weight-loss. The findings suggest that both PRO measures provide an opportunity to show treatment benefit in physical functioning, as a small change would represent a noticeable improvement in participants’ daily lives.

## Background

Nearly a third of the world’s population is now classified as living with overweight or obesity [1, 2] with worldwide prevalence tripling between 1974 and 2016 [3]. Obesity is a chronic disease associated with serious metabolic, psychological and physical sequelae, including type 2 diabetes (T2DM), cardiovascular disease, depression, sleep apnea, osteoarthritis, increased risk of cancer and more recently, Covid-19 [4–6]. Compounding these effects, obesity causes a significant reduction in health-related quality of life (HRQoL), including physical functioning [7, 8], ability to carry out normal activities of daily living (ADLs) [9, 10], emotional functioning [8] and bodily pain [11].

As HRQoL, including physical functioning, is an important concern for individuals living with obesity, it has been recommended that HRQoL be assessed as part of weight-management treatment and research [12]. Patient-reported outcome (PRO) measures are increasingly being used in clinical trials to evaluate HRQoL. Two PRO measures have been frequently used in prior weight-management studies: the acute 36-item Short Form Health Survey® (SF-36®) [13–15] and versions of the Impact of Weight on Quality of Life (IWQOL) measure, including the Lite-Clinical Trials Version^©^ (IWQOL-Lite-CT^©^) [16, 17].

The SF-36 is a generic PRO measure used to assess general health status. The measure consists of eight domains and provides two health component summary scores (physical and mental health) [13]. The physical health summary score is comprised of four domains: physical function, role-physical, bodily pain and general health. The mental health summary score is composed of vitality, social functioning, role-emotional and mental health. The physical function domain, the focus of the current study, is comprised of 10 items assessing activities completed during a typical day and how a participant’s health limits them in these activities. Each of the 10 items is assessed on a three-point Likert severity scale.

The IWQOL-Lite-CT version was designed for use in the context of clinical trials for adults living with overweight/obesity, both with and without T2DM, with the potential to support labeling claims for treatments of chronic weight-management. It consists of 20-items across two domains (physical and psychosocial); scores for a physical function composite, comprised of a subset of items in the physical domain, can also be obtained [16, 17].

Studies of lifestyle-based interventions and pharmacotherapy [18–20] have demonstrated improvements in several dimensions of HRQoL using various versions of the SF-36 and IWQOL. Physical functioning is the aspect of HRQoL that has consistently shown the most improvement across studies to-date [18]. In the Diabetes Prevention Program, a randomized controlled trial in over 3200 individuals with prediabetes (mean body mass index (BMI): 34 kg/m^2^) comparing intensive lifestyle intervention, metformin and placebo, the mean change from baseline compared to placebo over 3.2 years in the SF-36 physical function domain was 3.58 in the lifestyle group versus 0.13 in the metformin group (*p* < 0.01) [21].

Pharmacotherapy weight-loss trials have recently reported changes in physical function using both the SF-36v2 and different versions of IWQOL. The glucagon-like peptide-1 receptor agonist (GLP-1RA), liraglutide, yielded a statistically significant improvement in physical function score compared to placebo at 3 years (3.65 vs 2.18 on the SF-36v2 and 13.47 vs 8.99 on the IWQOL)[22]. More recently, the long-acting GLP-1RA, semaglutide has demonstrated statistically significant placebo-adjusted improvements of 1.5 to 1.8 on the SF-36v2 physical function score and 4.8 to 9.4 on the IWQOL-Lite-CT physical function score [23, 24].

Although these randomized controlled trials have shown statistically significant patient-reported improvement in physical function after weight-loss interventions, it is not clear if the magnitude of observed change is clinically meaningful within the populations studied. Some studies applied definitions of meaningful difference for SF-36 scores that were based on the general population or other disease states [21], rather than the population with overweight or obesity. Meaningful change thresholds have been proposed for individuals with overweight and obesity for the IWQOL-Lite-CT [25] and the IWQOL-Lite version [26] (an earlier iteration of the measure), but those thresholds were determined using statistical methods rather than incorporating qualitative patient insights.

Without an established threshold for what constitutes a meaningful change, it is difficult to interpret the potential therapeutic benefit of weight-loss interventions with regards to physical functioning. The findings from a preliminary evidence review of published literature suggested that further qualitative research is required to establish patient-perceived meaningful change thresholds for the SF36v2 and the IWQOL-Lite-CT in individuals living with overweight or obesity.

The most informative qualitative insights regarding meaningful change in physical function have yet to be derived from interviews of patients who have already experienced weight-loss in ongoing therapeutic trials. However, in obesity care, patient expectations prior to weight-loss have been shown to play an important role in perceived therapeutic benefit. Therefore, as a preliminary step towards understanding meaningful change in physical function, a qualitative interview study was conducted to explore the level of change on the SF-36v2 (acute) and IWQOL-Lite-CT that individuals living with overweight or obesity would *expect* to be meaningful and indicative of treatment success, and to examine how these expectations vary with different degrees of theoretical weight-loss [27–29]. This data can then be combined with qualitative and quantitative data from weight-loss trials to inform a more accurate threshold for meaningful change in physical function.

## Methods

Ninety-minute, semi-structured interviews were conducted across three regions in the United States (West, South, and Midwest).The target population of interest was individuals who would be candidates for anti-obesity pharmacotherapy, based on clinical characteristics. Inclusion criteria, therefore, included age ≥ 18 years, BMI ≥ 30 kg/m^2^ or BMI ≥ 27 kg/m^2^ with a weight-related comorbidity, stable weight for at least 3 months prior to screening, and at least one unsuccessful self-reported dietary effort to lose body weight. Those with prior or planned surgical treatment for obesity (including device-based therapies), recent treatment with prescription or over-the-counter weight-loss or weight gain-promoting medications, or history of psychiatric disorder or suicidal behavior were excluded.

Sampling quotas were used to ensure representation of adults with and without weight-related comorbidities (including T2DM, hypertension, dyslipidemia, obstructive sleep apnea or cardiovascular disease) in addition to ensuring representation of key demographic characteristics (including gender, age, race and ethnicity, employment status and education level). The study protocol was reviewed and approved by the New England Independent Review Board in September 2019 (ref: 120190277).

## Interview procedure

A step-wise approach was used in the interview to allow participants to first establish the meaning of key concepts (e.g., the smallest change that would be noticeable) and then apply those concepts within the context of each of the PRO measures (see Supplementary Material 1)*.*

### Conceptualizing meaningful change

Patient-perceived meaningfulness is a highly subjective measure, and expectations for meaningful change in physical function may vary according to the degree of weight-loss experienced. Moreover, weight-loss in therapeutic trials and clinical practice is often described as percent of total body fat, with 5%, 10% and 15% being frequently assessed endpoints or treatment targets. Since meaningfulness and percent weight-loss are not intuitive concepts for most patients, it was important for participants to first establish an individual understanding of these concepts before being asked to apply them to the SF-36v2 and IWQOL-Lite- CT version.

**Fig. 1:**
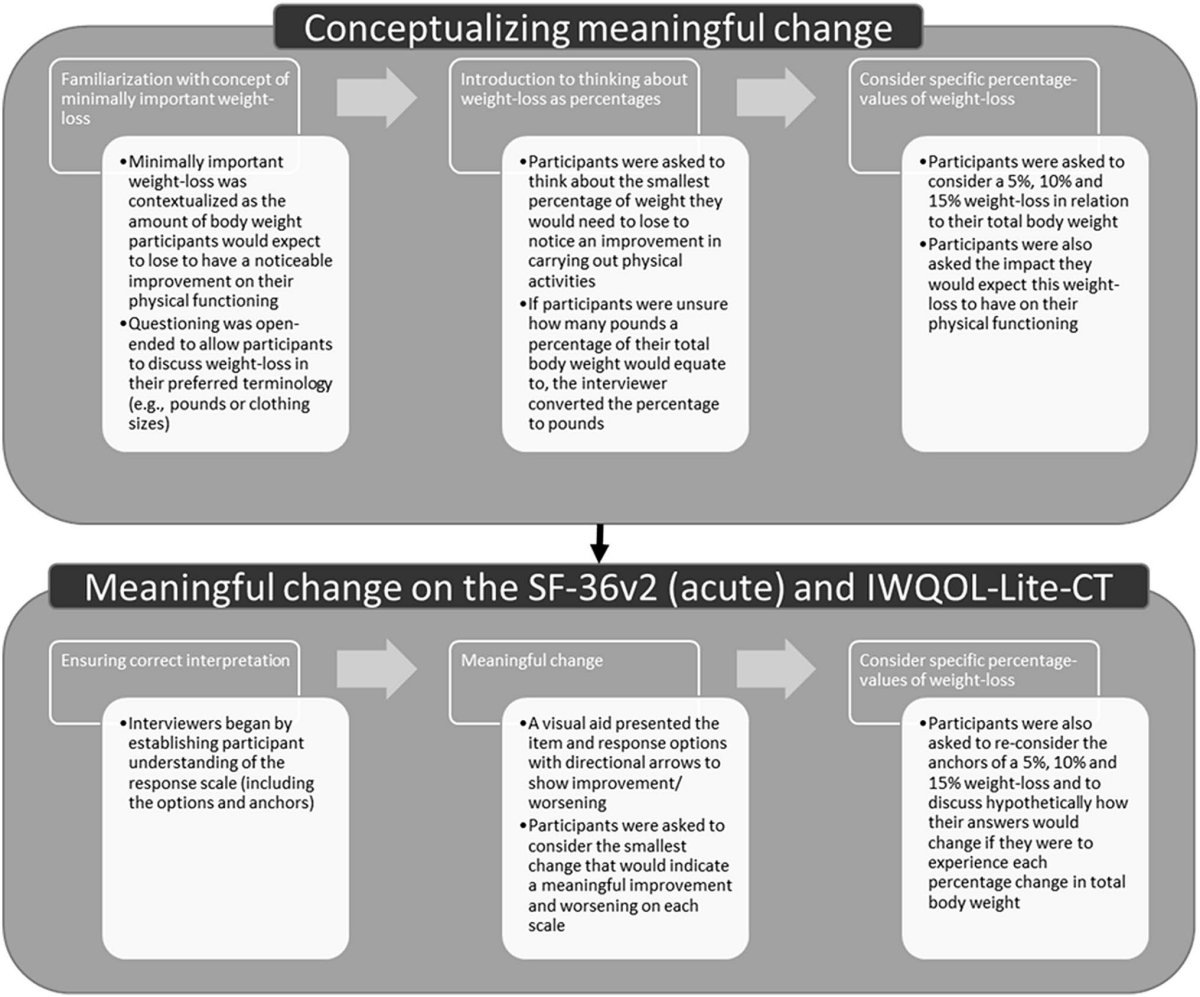
Overview of interview procedure: conceptualizing and defining meaningful change

Open-ended, concept elicitation techniques were used to provide non-biased, spontaneous insights into what a meaningful change in physical function would be for individuals (Fig. 1). This allowed participants to then discuss the amount of body weight they felt that they would need to lose to have a noticeable improvement on their physical functioning.

### Meaningful change on the SF-36v2 (acute) and IWQOL-Lite-CT

Cognitive interviewing techniques were used to establish participants’ understanding, interpretation and relevance of the PRO measures, and to determine thresholds for meaningful improvements or worsening on example items with different response scales in each measure [30]. A step-wise approach was utilized to explore meaningful change on each PRO (Fig. 1) [31].

Selected items from the physical function domain on each PRO were used to explore meaningful change with participants. The item selected to explore meaningful change thresholds on the SF-36v2 (acute) was “Item 3f: Bending, kneeling or stooping” as this was expected to be a highly relevant item across the sample based on prior literature. Response options for Item 3f are on a 3-point Likert scale (assessing severity of impacts: “Yes, limited a lot”, “Yes, limited a little” or “No, not limited at all”). For assessing meaningful improvement, participants were asked to consider ‘yes, limited a lot’ as their starting point and to select the smallest change from this point which would indicate a meaningful improvement in bending, kneeling or stooping.

To explore the two different response scales used in the IWQOL-Lite-CT, “Item 2: I get tired or winded walking up one flight of stairs” and “Item 16: I am not as physically active as I would like to be” were selected. For Item 2, response options are on a 5-point Likert scale (assessing frequency of impacts) ranging from ‘Never’ to ‘Always’. For assessing both meaningful improvement and worsening, participants were asked to consider ‘sometimes’ as the starting point and to select the smallest change from this point which would indicate a meaningful improvement or worsening. For Item 16, response options are on a 5-point Likert scale (assessing how true each statement is) ranging from ‘Not at all true’ to ‘Completely true’. For both meaningful improvement and worsening, participants were asked to consider ‘moderately true’ as the starting point and to select the smallest change from this point which would indicate a meaningful improvement or worsening.

As part of the PRO meaningful change discussion, participants were also asked to re-consider the anchors of a 5%, 10% and 15% weight-loss and to discuss hypothetically how their answers would change if they were to experience each percentage change in total body weight. This will allow future researchers to understand whether observed physical function improvement with weight-loss in clinical trials meets expectations or not.

### Sub-group comparisons

The participants were categorized into one of three sub-groups, specified a priori*,* to determine if there were any notable differences in the experience of living with obesity. This analysis was exploratory, with no pre-specified hypotheses. The sub-groups comprised of individuals with BMI ≥ 27 kg/m^2^ with T2DM, BMI ≥ 30 kg/m^2^ without T2DM, but with at least one weight-related comorbidity^1^ and those with a BMI ≥ 30 kg/m^2^ without T2DM or any of the weight-related comorbidities. Qualitative descriptions were explored across the sub-groups and any differences were noted. As part of the PRO meaningful change analysis, the mean point change indicative of a meaningful improvement and worsening were compared across the sub-groups.

## Results

Thirty-three participants were interviewed (Table 1). The sample was equally split across male (*n* = 17, 52%) and female (*n* = 16, 48%) participants, with a mean age of 45 years old. Most participants were educated to high school diploma or equivalent (*n* = 14, 42%), college/associate’s degree (*n* = 7, 21%) or Bachelor’s degree (*n* = 7, 21%). Geographical diversity within the US was also achieved across the South (*n* = 8, 24%), Midwest (*n* = 12, 36%), and West (*n* = 13, 39%).

**Table 1:** Participant clinical characteristics and demographics

Clinical and demographic characteristics	Total (*N* = 33) *n* (%)	Individuals with overweight or obesity (BMI ≥ 27 kg/m^2^) with T2DM (*N* = 12) *n* (%)	Individuals with obesity (BMI ≥ 30 kg/m^2^) without diabetes, AND with ≥ 1 weight-related comorbidity (*N* = 12) *n* (%)	Individuals with obesity (BMI ≥ 30 kg/m^2^) without diabetes or weight-related comorbidities (*N* = 9) *n* (%)
Current weight (kg)	109.2 (104.3)	102.5 (102.1)	111 (108.7)	114.1 (104.3)
Mean (Median) [Range]	[73.9–170.1]	[73.9–148.8]	[88–140.2]	[84.4–170.1]
Current BMI (kg/m^2^)	37.6	35.5	39.0	38.4
Mean [Range]	[27.4–56.6]	[27.4–49.9]	[30.0–56.6]	[30.0–50.9]
Comorbidities^a^				
T2DM Hypertension Obstructive sleep apnea Dyslipidemia Cardiovascular disease	12 (36%) 11 (33%) 6 (18%) 3 (9%) 3 (9%)	12 (100%) 3 (25%) 1 (8%) 1 (8%) 2 (17%)	N/A^±^ 8 (67%) 5 (42%) 2 (17%) 1 (8%)	N/A^±^ N/A N/A N/A N/A
Weight-loss treatment history				
Currently on treatment Previously received treatment Treatment naive	1 (3%) 3 (9%) 29 (88%)	0 0 12 (100%)	1 (8%) 1 (8%) 10 (83%)	0 2 (22%) 7 (78%)
Sex				
Male Female	17 (52%) 16 (48%)	8 (67%) 4 (33%)	6 (50%) 6 (50%)	3 (33%) 6 (67%)
Age, years	45 (43)	51 (49)	42 (40)	43 (44)
Mean (Median) [Range]	[19–81]	[36–81]	[19–75]	[24–56]
Highest level of education				
High school, but no diploma High school diploma or equivalent College or associate’s degree Bachelor’s degree Some graduate/post-graduate work Graduate/post-graduate degree Other (Vocational qualification)	3 (9%) 14 (42%) 7 (21%) 7 (21%) 1 (3%) 0 1 (3%)	2 (17%) 4 (33%) 4 (33%) 2 (17%) 0 0 0	1 (8%) 5 (42%) 3 (25%) 2 (17%) 1 (8%) 0 0	0 5 (56%) 0 3 (33%) 0 0 1 (11%)

Weight was reported by participants in pounds, but has been converted into kg

^a^Clinicians may have selected multiple responses for each participant; ± Not applicable due to the sub-group eligibility criteria (e.g., Obesity without diabetes or weight-related comorbidities)

The mean weight of the sample was 109 kg (range 74–170 kg). The mean BMI was 37.6 kg/m^2^ (range 27.4–56.6 kg/m^2^). Of those participants who had weight-related comorbidities, fifteen reported only one (*n* = 15/24, 63%) weight-related comorbidity. Comorbid conditions included T2DM (*n* = 12/24, 50%), hypertension (*n* = 11/24, 46%) and obstructive sleep apnea (*n* = 6/24, 25%). The majority of the sample (*n* = 29/33, 88%) were treatment naïve to anti-obesity medications and none had previously undergone bariatric surgery.

### Conceptualizing meaningful change

Participants were first familiarized with the concept of meaningful weight-loss and asked to discuss the percentage of their body weight they would expect to lose to have a noticeable improvement on their physical functioning (interview guide excerpt provided in Supplementary Material 1).

The majority of participants chose to respond to the interviewer questions in pounds (*n* = 22), but a number of participants (*n* = 7) offered answers in percentages. Two participants had difficulty with percentages and a further two participants did not answer.

Then, the interviewer asked the participants to specifically visualize a 5%, 10% and 15% change in body weight. Since percent weight-loss is not an intuitive measure for patients, each percentage change was also provided in both pounds (lbs) and kilograms (kgs) if requested by the participant, based on each individual’s own weight.

### “Would a 5% weight-loss be noticeable to you?”

Over two-thirds of participants (23/33, 70%) felt that a 5% weight-loss would be noticeable (Table 2). When discussing the difference a 5% weight-loss would make, participants expected they would experience increased energy (*n* = 7)/stamina (*n* = 2) and that they would be more active (*n* = 3). In terms of physical functioning, participants expected a noticeable improvement in their ability to stand (*n* = 2) and to walk longer distances (*n* = 2). Additionally, participants expected a physiological benefit to a 5% weight-loss, for example, less pain while completing daily activities (*n* = 3) and improved breathing (*n* = 2). Ten participants (10/33, 30%) did not feel 5% weight-loss would be noticeable enough to make a difference in performing daily activities.

### “Would a 10% weight-loss be noticeable to you?”

All participants (33/33, 100%) reported that they expected that a 10% weight-loss would be noticeable (Table 2). When describing a 10% weight-loss, participants expected to experience increased energy (*n* = 15), be more agile/mobile (*n* = 5) and/or be more physically active (*n* = 4). Participants felt that a 10% weight-loss may improve their ability to walk, either for longer distances or at a quicker pace (*n* = 5), hike (*n* = 2) and complete household chores (*n* = 2). One participant, who had previously used a walker, mentioned they would expect to be able to move without assistance. Similar to a 5% weight-loss, participants expected some additional physiological benefits including less pain (*n* = 3) and improved breathing (*n* = 2).

**Table 2:** Participant perceptions of meaningful weight-loss

% Weight-loss	Noticeable? (*N* = 33)		Participant quotes
	Y	N	
5%	*n* = 23 (70%)	*n* = 10 (30%)	**Yes, noticeable:** “I think that with some activities that I might want to do, I might be able to walk to the end of the block. I think that would give me back some of my mobility” (Female, 44, BMI: 47.3 kg/m^2^) “… Being able to bend over and do stuff a little bit better” (Male, 32, BMI: 50.9 kg/m^2^)
			**No, not noticeable:** “No, I don’t think so. It would be nice, but I don’t think it’s noticeable or would make a difference” (Male, 43, BMI: 40.2 kg/m) “I don’t think that’s enough for me to lose really to be noticeable” (Male, 61, 34.2 kg/m)
10%	*n* = 33 (100%)	*n* = 0 (0%)	**Yes, noticeable:** “It would make a big difference. I think like I said, I would be more agile. I would have more energy to do things. I wouldn’t feel as tired. More energy I think”(Female, 50, BMI: 35.2 kg/m^2^) “And also, the activities you do. I mean, you’re more—you want to do them. Like before, you fretted doing cutting the grass; now you look forward to cutting the grass” (Male, 53, BMI: 42.8 kg/m^2^) “I could probably wear some of the clothes that I have hiding in my closet that I haven’t worn in a while […] And then definitely my energy level would be that much higher, you know […] I can probably do more planking and more activities at the gym” (Female, 39, BMI: 40.0 kg/m)
15%	*n* = 33 (100%)	*n* = 0 (0%)	**Yes, noticeable:** “Oh, just like I said before, the more weight I lose, the more flexibility I have and the less pain on the knees and on the back.” (Male, 54, BMI: 27.5 kg/m^2^) “Yeah. I would be able to go up some stairs without losing breath. I wouldn't stop halfway up the stairs. I only have 15 stairs. My daughter’s at the top and I'm in the middle. I’m catching my breath. It would just be a really big change. I would be able to do things that I can’t now”(Female, 20, BMI: 33.9 kg/m^2^) “I'd probably be able to walk. I'd probably be able to bend, throw, do whatever” (Female, 45, BMI: 39.9 kg/m^2^) “Probably be able to go out hiking and do more things, like to go amusement park and ride a rollercoasters, because those rollercoasters are not big people friendly” (Male, 32, BMI: 50.9 kg/m^2^)

### “Would a 15% weight-loss be noticeable to you?”

All participants (33/33, 100%) reported that a 15% weight-loss would be noticeable (Table 2). Participants expected improvements in climbing the stairs (*n* = 2) and exercising (*n* = 2) if they experienced a 15% weight-loss. One participant reported they would expect to be able to care for themselves and not need to rely on others for assistance when moving around. Compared to a 5% or 10% weight-loss, twice as many participants expected a reduction in pain (*n* = 6) with a 15% weight-loss and the same number expected improved breathing (*n* = 2).

#### Sub-group comparisons

For a theoretical 5% body weight-loss, individuals ‘without T2DM but with at least one other weight-related comorbidity’ were more likely to expect the change to be noticeable (*n* = 10/12, 83%) than those ‘with T2DM’ (*n* = 8/12, 67%) or those ‘without T2DM or any other weight-related comorbidities’ (*n* = 5/8, 62.5%).

### Meaningful change: SF-36v2 (acute)®

Thirty-one participants completed the meaningful change task for Item 3f (Bending, kneeling or stooping) of the SF-36v2 (acute). The interviewer began the task by determining that the participants understood the underlying response scale [31], which all participants appeared to.

A 1-point change on Item 3f was considered the smallest meaningful improvement by the majority of participants (*n* = 28) from a suggested starting point of “Yes, limited a lot”. Similarly, a 1-point change was considered indicative of meaningful worsening for the majority (*n* = 20) when starting from “No, not limited at all”.

When considering how their ability to bend, kneel or stoop may change with a 5% or 10% total body weight-loss, participants expected that their response to Item 3f would likely improve by 1-point (5% weight-loss, *n* = 18; 10% weight-loss, *n* = 16). However, when imagining a 15% total body weight-loss, participants (*n* = 21) expected a larger point change of 2-points (Table 3).

**Table 3:** Perceptions of meaningful change on the SF-36v2 (acute) and the IWQOL-Lite-CT

SF-36v2 (acute)—Item 3f—bending, kneeling or stooping (Severity scale)				
Change from baseline ‘Yes, limited a lot’	Point change considered meaningful			Mean^a^
	0 ‘Yes, limited a lot’	+ 1 ‘Yes, limited a little’	+ 2 ‘No, not limited at all’	
Smallest meaningful improvement (*n* = 31)	0	28	3	1.1
5% weight-loss (*n* = 31)	11	18	2	0.7
10% weight-loss (*n* = 31)	4	16	11	1.2
15% weight-loss (*n* = 31)	3	7	21	1.6

Change from baseline ‘No, not limited at all’	Point change considered meaningful			Mean^a^
	0 ‘No, not limited at all’	− 1 ‘Yes, limited a little’	− 2 ‘Yes, limited a lot’	
Smallest meaningful worsening (*n* = 29)	0	19	10	1.3

IWQOL-Lite-CT Item 2—Tired or winded walking up one flight of stairs (Frequency scale)				
Change from baseline ‘Sometimes’	Point change considered meaningful			Mean^a^
	0 ‘Sometimes’	− 1 ‘Rarely’	− 2 ‘Never’	
Smallest meaningful improvement (*n* = 31)	0	29	2	1.1
5% weight-loss (*n* = 31)	11	19	1	0.7
10% weight-loss (*n* = 31)	4	16	11	1.2
15% weight-loss (*n* = 31)	2	9	20	1.6

Change from baseline ‘Sometimes’	Point change considered meaningful			Mean^a^
	0 ‘Sometimes’	+ 1 ‘Usually’	+ 2 ‘Always’	
Smallest meaningful worsening (*n* = 30)	0	26	4	1.1

IWQOL-Lite-CT—Item 16 – Not as physically active as I would like to be (Truth scale)				
Change from baseline ‘Moderately true’	Point change considered meaningful			Mean^a^
	0 ‘Moderately true’	− 1 ‘A little true’	− 2 ‘Not at all true’	
Smallest meaningful improvement (*n* = 29)	0	27	2	1.1
5% weight-loss (*n* = 30)	12	18	0	0.6
10% weight-loss (*n* = 31)	4	17	10	1.2
15% weight-loss (*n* = 30)	0	11	19	1.6

Change from baseline ‘Moderately true’	Point change considered meaningful			Mean^a^
	0 ‘Moderately true’	+ 1 ‘Mostly true’	+ 2 ‘Completely true’	
Smallest meaningful worsening (*n* = 29)	0	20	9	1.3

^a^Mean rounded to one decimal place

#### Sub-group comparisons

The findings were broadly consistent across the three sub-groups (Table 4). When considering a 10% and a 15% weight-loss, the mean point score considered a meaningful change was higher for individuals ‘without T2DM but at least one other weight-related comorbidity’ than those ‘with T2DM’ and those ‘without T2DM or any other weight-related comorbidities’.

**Table 4:** Perceptions of meaningful change on the SF-36v2 (acute) the IWQOL-Lite-CT sub-group comparisons

SF-36v2 (acute)—Item 3f—bending, kneeling or stooping (Severity scale)			
Change from baseline ‘Yes, limited a lot’	Mean point change considered meaningful^a^		
	BMI ≥ 27 kg/m^2^ with T2DM	BMI ≥ 30 kg/m^2^ without T2DM but with other comorbidities	BMI ≥ 30 kg/m^2^) without T2DM or any other comorbidities
Smallest meaningful improvement (*n* = 31)	1.0	1.2	1.1
Theoretical expectation: 5% weight-loss (*n* = 31)	0.6	0.9	0.6
Theoretical expectation: 10% weight-loss (*n* = 31)	0.9	1.7	1.0
Theoretical expectation: 15% weight-loss (*n* = 31)	1.5	1.7	1.6
*Change from baseline ‘No, not limited at all’*			
Smallest meaningful improvement (*n* = 29)	1.2	1.4	1.4

IWQOL-Lite-CT Item 2—Tired or winded walking up one flight of stairs (Frequency scale)			
Change from baseline ‘Sometimes’	Mean point change considered meaningful^a^		
	BMI ≥ 27 kg/m^2^ with T2DM	BMI ≥ 30 kg/m^2^ without T2DM but with other comorbidities	BMI ≥ 30 kg/m^2^) without T2DM or any other comorbidities
Smallest meaningful improvement (*n* = 31)	1.0	1.1	1.1
Theoretical expectation: 5% weight-loss (*n* = 31)	0.5	0.9	0.7
Theoretical expectation: 10% weight-loss (*n* = 31)	1.1	1.5	1.0
Theoretical expectation: 15% weight-loss (*n* = 31)	1.5	1.9	1.3
*Change from baseline ‘No, not limited at all’*			
Smallest meaningful worsening (*n* = 30)	***1.1***	***1.1***	***1.2***

IWQOL-Lite-CT—Item 16—Not as physically active as I would like to be (Truth scale)			
Change from baseline ‘Moderately true’	Mean point change considered meaningful^a^		
	BMI ≥ 27 kg/m^2^ with T2DM	BMI ≥ 30 kg/m^2^ without T2DM but with other comorbidities	BMI ≥ 30 kg/m^2^) without T2DM or any other comorbidities
Smallest meaningful improvement (*n* = 29)	1.0	1.2	1.0
Theoretical expectation: 5% weight-loss (*n* = 30)	0.4	0.9	0.4
Theoretical expectation: 10% weight-loss (*n* = 31)	1.0	1.5	1.0
Theoretical expectation: 15% weight-loss (*n* = 30)	1.4	2.0	1.5
*Change from baseline ‘Moderately true’*			
Smallest meaningful worsening (*n* = 29)	1.2	1.4	1.3

^a^Mean rounded to one decimal place

### Meaningful change: IWQOL-Lite-CT^©^ (frequency response scale)

Thirty-one participants completed the meaningful change task for Item 2 (Tired or winded walking up one flight of stairs) of the IWQOL-Lite-CT, which utilized a frequency response scale. All participants appeared to understand the response scale before discussing meaningful improvements/worsening.

A 1-point change on Item 2 was considered the smallest meaningful improvement by the majority of participants (*n* = 29) from the suggested starting point of “sometimes”. Similarly, a 1-point change was considered indicative of meaningful worsening for the majority (*n* = 26) from the same starting point.

When considering how their ability to walk up one flight of stairs (without feeling tired or winded) may change with a 5% or 10% total body weight-loss, participants expected that their response to Item 2 would improve by 1-point (5% weight-loss, *n* = 19; 10% weight-loss, *n* = 16). However, when imagining a 15% total body weight-loss, participants (*n* = 20) expected their response to improve by 2-points (Table 3).

### Meaningful change: IWQOL-Lite-CT^©^ (‘Truth’ response scale)

Thirty-one participants completed the meaningful change task for Item 16 (Not as physically active as I would like to be) of the IWQOL-Lite-CT, which utilized a truth response scale. All participants appeared to understand the response scale before discussing meaningful improvements/worsening.

A 1-point change on Item 16 was considered the smallest meaningful improvement by the majority of participants (*n* = 27) from the suggested starting point of “moderately true”. Similarly, a 1-point change was considered indicative of meaningful worsening for the majority (*n* = 20) from the same starting point.

When considering how their ability to be physically active may change with a 5% or 10% total body weight-loss, participants expected that their response to Item 16 would likely improve by 1-point (5% weight-loss, *n* = 18; 10% weight-loss, *n* = 17). However, when imagining a 15% total body weight-loss, participants (*n* = 19) expected their response to improve by 2-points (Table 3).

#### Sub-group comparisons

The findings were broadly consistent across the three sub-groups on both IWQOL-Lite-CT response scales (Table 4). When considering a theoretical 5%, 10% and a 15% weight-loss, the mean point scores considered a meaningful change were higher for individuals ‘without T2DM but at least one other weight-related comorbidity’ than those ‘with T2DM’ and those ‘without T2DM or any other weight-related comorbidities’.

## Discussion

This study qualitatively explored perceptions of meaningful improvement in physical function as assessed by two PRO measures in individuals with overweight/obesity, and expectations of physical function improvement with varying degrees of weight-loss. Findings suggest nearly two-thirds of people living with overweight or obesity expect a 5% total body weight-loss to be enough to yield some benefit in physical functioning. Meanwhile, all participants expected a weight-loss of at least 10% to provide a meaningful and noticeable improvement to their physical functioning, such as participation in more strenuous or varied physical activities. This logical trend of increasing improvement in physical functioning expected with an increased weight-loss is strong support that participants were able to understand the meaningful change tasks and provide examples of these expected improvements from their daily lives.

Previous research has highlighted a disparity between patients’ expectations from weight-loss treatment and provider-directed weight-loss goals [32–34]. Individuals with overweight or obesity expect to reach an often unrealistic “goal weight” while providers aim for a more achievable 5–10% initial weight-loss that yields metabolic benefits and cardiovascular risk reduction. The findings from this study suggest that participants anticipated a potential improvement in physical function with 5–10% weight-loss, and as such, discussions of potential physical function improvement with 5–10% weight-loss may be used by providers to help align patients on initial weight-loss goals [27–29].

While the majority of participants in the current study reported that a 5–15% weight-loss would be noticeable and likely to provide a meaningful improvement to their physical functioning, there was a lack of understanding by many participants regarding how much of their total body weight the percentages would equate to until the interviewer converted this into absolute units (pounds). This suggests that individuals may not be able to conceptualize the idea of percentage reductions in body weight and therefore it may be more appropriate to discuss weight-loss with patients in terms of pounds or kilograms in clinical practice.

The data from cognitive debriefing provide an initial insight into the point change that may represent a meaningful improvement or worsening at a single item-level on the SF-36v2 (acute) physical function domain and the IWQOL-Lite-CT from the participants’ perspective. The majority of participants reported that a 1-point score change on select items would represent a meaningful improvement in their ability to carry out daily activities, such as being able to walk further or climb more flights of stairs, and would be indicative of treatment success. This item-level 1-point patient-perceived meaningful change threshold may be built upon in future research to define the domain-level minimal clinically important difference (MCID) in physical function resulting from weight-loss. Prior estimates of an MCID for the IWQOL-Lite-CT [25] were based on quantitative anchor-based methods at the domain-level, and did not incorporate qualitative patients’ perspectives. Our qualitative findings can be combined with quantitative clinical trial data to inform a more clinically relevant MCID.

Overall, the findings from this study were broadly consistent across the pre-defined overweight/obesity sub-groups. In the cognitive debriefing meaningful change tasks, the sample of individuals ‘without diabetes but at least one weight-related comorbidity’ typically noted that they would expect to see a larger point change with a 10% or 15% total body weight-loss than individuals ‘with diabetes’ and individuals ‘without T2DM and any other weight-related comorbidities’. Additionally, this sub-group also expected that a 5% weight-loss would be more noticeable when compared with the other two sub-groups. Therefore, expectations of weight-loss-induced physical and health benefits may vary according to subgroup. Weight-related comorbidities (e.g., hypertension, dyslipidemia, obstructive sleep apnea or cardiovascular disease) may lead to individuals expecting more substantial changes to their physical functioning following successful weight-loss.

This study does have some limitations that should be noted. The nature of qualitative research means that findings are based on a relatively small sample (*n* = 33) of individuals living with overweight/obesity in the United States. Although ethnic, racial and geographic diversity was achieved in the sample, results should still be generalized to a broader, cross-cultural population with caution. Each overweight/obesity sub-group in this study only included up to 12 participants, and therefore any differences among groups noted in this research should be considered exploratory and indicative rather than confirmatory. The discussions regarding 5–15% total body weight-loss were theoretical, with questions around *expected* weight-loss not *experienced* weight-loss, and therefore may have been difficult to conceptualize for any individuals who had not experienced the level of weight-loss in question. Exit or in-trial interviews will be beneficial to explore the perspectives of participants who have achieved these percentage weight-loss thresholds. The PRO measures used in this study had relatively small response scales (3-point and 5-point Likert scales). To facilitate the cognitive debriefing meaningful change tasks, participants were asked to start at the midpoint of each response scale for the IWQOL-Lite-CT and at the higher or lower end of the response scale for SF-36v2, to allow comment on both improvement and worsening on the scale. As such, the results of this activity were somewhat limited by the number of possible responses.

The findings from this study provide an in-depth exploration of what individuals living with overweight and obesity consider to be a meaningful change in physical functioning; however, this evidence should not be taken in isolation. This qualitative evidence should be triangulated with anchor and distribution-based statistical analyses to establish values for minimally important differences on each PRO measure. Future research should then explore whether individuals’ expectations for physical functioning benefits are met once weight-loss is achieved.

## Conclusion

In summary, individuals living with overweight and obesity reported that they expect 5–15% body weight-losses to be noticeable and provide meaningful improvements to their physical functioning, with 10% or higher weight-loss yielding the most consistent results. The findings from the cognitive debriefing tasks indicate that both SF-36v2 (acute) and IWQOL-Lite-CT PRO measures provide strong opportunity to demonstrate treatment benefit in relation to physical functioning and people with overweight/obesity expect a 1-point change on the response scale to represent a noticeable improvement in their daily lives.

## Supplementary Information

Below is the link to the electronic supplementary material.

Supplementary file1 (PDF 173 kb)

## Data Availability

Not applicable.

## Footnotes

1 Weight-related comorbidities were specified as hypertension, dyslipidemia, obstructive sleep apnea or cardiovascular disease (treated or untreated).
