# Supplementary material for: A qualitative study to examine meaningful change in physical function associated with weight-loss
Source: Qual Life Res. 2022 Jul 22;32(5):1329–40. doi: 10.1007/s11136-022-03191-2 (PMC9305034; doi:10.1007/s11136-022-03191-2)
Supplement: Supplementary file 1 — Supplementary file1 (PDF 173 kb) [file 11136_2022_3191_MOESM1_ESM.pdf]

Supplementary material 1: Interview guide excerpt

Theoretical expectations:

I will now ask you a few questions to help us understand what you would consider to be a meaningful change to your weight to achieve noticeable improvements in your daily life/activities.

1) **As a % of your total body weight, what is the smallest amount of weight would you need to lose to notice an improvement in carrying out physical activities?**
   a) What are some activities that would be improved?
2) **Would a 5% weight loss be noticeable to you?**
   a) **If yes:** What difference would this make in your daily activities?
3) **Would a 10% weight loss be noticeable to you?**
   a) **If yes:** What difference would this make in your daily activities?
4) **Would a 15% weight loss be noticeable to you?**
   a) **If yes:** What difference would this make in your daily activities?

Meaningful change: SF-36v2 (Item 3f)

We are going to complete this task thinking of question 3f on the first questionnaire. The question asks if your health limits you in certain activities and for this exercise we're going to think about 'bending, kneeling or stooping'.

Yes, limited a lot	Yes, limited a little	No, not limited at all

1) **You selected the answer [X] when answering this question. Can you describe what this feels like?**
2) **Let us imagine that you selected the answer 'Yes, limited a lot'. What would be the smallest change you would be able to notice and consider a meaningful improvement?**
3) **If you were to answer this question thinking about a 5% weight loss, how would your answer change from 'yes, limited a lot'?**
   a) How noticeable would this change be?
4) **If you were to answer this question thinking about a 10% weight loss, how would your answer change from 'yes, limited a lot'?**
   a) How noticeable would this change be?

5) **If you were to answer this question thinking about a 15% weight loss, how would your answer change from 'yes, limited a lot'?**
   a) How noticeable would this change be?

6) **How about if things got worse? From 'No, not limited at all', what would be the smallest change you would notice and consider a worsening?**
   a) How noticeable would this change be?

Meaningful change: IWQOL-Lite-CT (Item 2)

Here is a frequency scale, with 'Never' being the least frequent and 'Always' the most frequent. We are going to complete this task thinking of question 2 of the second questionnaire 'I get tired or winded walking up one flight of stairs'.

Never	Rarely	Sometimes	Often	Always

7) **You selected the answer [X] when answering this question. Can you describe what this feels like?**

8) **Let us imagine that you selected the answer 'sometimes'. What would be the smallest change you would be able to notice and consider a meaningful improvement?**

9) **If you were to answer this question thinking about a 5% weight loss, how would your answer change from 'sometimes'?**
   a) How noticeable would this change be?

10) **If you were to answer this question thinking about a 10% weight loss, how would your answer change from 'sometimes'?**
   a) How noticeable would this change be?

11) **If you were to answer this question thinking about a 15% weight loss, how would your answer change from 'sometimes'?**
   a) How noticeable would this change be?

12) **How about if things got worse? From sometimes, what would be the smallest change you would notice and consider a worsening?**
   a) How noticeable would this change be?
